# Focal hyperintensity in the dorsal brain stem of patients with cerebellopontine angle tumor: A high-resolution 3 T MRI study

**DOI:** 10.1038/s41598-018-19232-1

**Published:** 2018-01-17

**Authors:** Hirotaka Yamamoto, Atsushi Fujita, Taichiro Imahori, Takashi Sasayama, Kohkichi Hosoda, Ken-ichi Nibu, Eiji Kohmura

**Affiliations:** 1 Department of Neurosurgery, Kobe University Graduate School of Medicine, Kobe, Japan; 2 Department of Otolaryngology-Head & Neck Surgery, Kobe University Graduate School of Medicine, Kobe, Japan

## Abstract

Focal hyperintensity (FHI) in the dorsal brain stem on T2-weighted images of patients with cerebellopontine angle (CPA) tumor was thought to indicate degeneration of the vestibular nucleus and to be specific to vestibular schwannoma. The purpose of this study was to evaluate FHI by using high-resolution 3 Tesla magnetic resonance imaging (3 T MRI) and the relation to clinical characteristics. We retrospectively reviewed the clinical data and MRI of 45 patients with CPA tumors (34 vestibular schwannomas and 11 other tumors). FHI in the dorsal brain stem was found in 25 (55.6%) patients (20 vestibular schwannomas and 5 other tumors). For the vestibular schwannomas, the factors contributing to positive FHI were age (p = 0.025), max CPA (p =  < 0.001), hearing ability (P = 0.005), and canal paresis (p =  < 0.001) in the univariate analysis. Multivariate regression analysis showed that max CPA (p = 0.029) was a significant factor of positive FHI. In other CPA tumors, these factors were not significant predictors. With the use of 3 T MRI, FHI was observed more frequently than previously reported. Our results suggest that FHI is not a specific indicator of vestibular schwannoma and is related to not only vestibular function but also other factors.

## Introduction

The cerebellopontine angle (CPA) cistern is a subarachnoid space containing many cranial nerves and vessels, and is bounded by the brain stem, the cerebellum and the petrous bone^1^. The majority of tumors in this space are vestibular schwannomas, which are often large and involve the cranial nerves and compress the brain stem^2,3^. In 2006, Okamoto reported that some patients with vestibular schwannoma showed a tiny area of hyperintensity in the dorsal brain stem on T2-weighted images^4^. This area was observed in 14.6% of the patients with vestibular schwannomas larger than 2 cm, and was not found in patients with other kinds of CPA tumors. They concluded that the presence of this area of hyperintensity indicated degeneration of the vestibular nucleus.

The vestibular nucleus consists of four separate nuclei which form a complex located towards the most lateral aspect of the floor of the fourth ventricle^5^. While the location of hyperintensity in the dorsal brain stem anatomically almost coincides with the vestibular complex, little else is known about it. The purpose of this study was to examine the focal hyperintensity (FHI) lesion of the dorsal brain stem with the aid of high-resolution 3 Tesla magnetic resonance imaging (3T-MRI) and to analyze its relationship with clinical features. Our hypothesis is that if the finding of FHI is related to the degeneration of nucleus, the correlation with clinical characteristics of these nucleus would be provided.

## Methods

This retrospective study is in accordance with the Declaration of Helsinki and International Council for Harmonisation/Good Clinical Practice guidelines. The analysis was approved by the Ethics Committee of the Kobe University School of Medicine, Japan. The respective institutional review boards approved this retrospective study and informed consent was obtained from all subjects. Patients’ records and information were anonymized and de-identified prior to the analysis.

### Subjects

After installation of high-field MRI, we applied MR cisternography for all preoperative patients with posterior fossa tumors^6^. This study included 45 consecutive patients with a CPA tumor (21 men and 24 women; mean age, 49.0 years; age range, 16–74 years) who underwent preoperative examination by 3T-MRI between May 2007 and March 2012. All cases were operated on by the senior surgeon (EK) and the origin of the tumors of all patients was determined from the surgical findings. The histopathological diagnoses were: vestibular schwannoma (34 patients; 76%), meningioma (5; 11%), non-vestibular schwannoma (4; 9%), epidermoid tumor and papilloma (1 each; 2%). Patients with neurofibromatosis were excluded from this study. At the same periods, five normal volunteers (4 men and 1 woman, 29–36 years old) were included for the control subjects. The clinical data of these cases were evaluated retrospectively collected and analyzed.

### MR imaging protocol

MR images were obtained with a 3 T clinical scanner (Intera Achieva; Philips, Eindhoven, The Netherlands). In our institute, 3-dimensional driven equilibrium (3D-drive) and balanced fast-field echo (bFFE) are performed in addition to the routine MRI sequences for the patients with the posterior cranial fossa lesion. The sequence parameters used for bFFE scans were as following: TR 7.3ms, TE 3.6 ms, FOV 150 × 150 mm, 35 slice with 0.7 mm slice thickness, in plane matrix size 256 × 256, reconstructed into 512 × 512 resulting 0.29 × 0.63 × 0.63 mm voxel size, and the acquisition time was 2 minutes 20 seconds. 3D-drive was used with the following parameters: TR 1500ms, TE 250ms, FOV 160 × 160 mm, 50 slice with 0.7 mm slice thickness, in plane matrix size 256 × 256 with 0.63 × 0.63 × 0.7 mm voxel size, and the acquisition time was 4 minutes 30 seconds. We used a contrast enhanced bFFE (balanced fast-field echo) technique to focus on precise visualization of the relation of the tumor to the cranial nerves. To enhance contrast between tumor and neighboring structures, we intravenously injected 0.1 mmol/kg Gd-diethylenetriamine pentaacetic acid (Gd-DTPA). Besides obtaining information necessary for successful tumor removal, we examined intensity changes within the dorsal brain stem.

### Evaluation of MR imaging

The presence of FHI in the dorsal brain stem was evaluated by two board-certified neurosurgeons with 20 and 12 years of experience independently and the final decision was reached by consensus. In each patients with FHI, we also evaluated whether the FHI include the anatomical structures based on the anatomical atlas Cytoarchitecture of the Human Brain Stem^7^. We considered characteristic structures such as floor of the fourth ventricle, median sulcus, inferior cerebellar peduncle, and olive as anatomical index in axial bFFE scans and compared these with the atlas slice by slice and defined the involved structures by consensus of two reviewers. Based on anatomical atlas, we defined which nucleus was involved among four vestibular nuclei, i.e., superior vestibular nucleus (SVNc), medial vestibular nucleus (MVNc), lateral vestibular nucleus (LVNc), and inferior vestibular nucleus (IVNc). In cases with large tumor (≥4 cm), because the distortion of brain stem made it difficult to compare with the atlas, we used postoperative imaging obtained more than six months after operation for the evaluation of the precise localization. Measurement of tumor size was based on max CPA, which is the maximum diameter of the portion of the tumor within the CPA cistern^8^.

### Otolaryngological examination

Hearing function was assessed by means of a pure-tone audiogram with a range up to 115 dB. Equilibrium function was evaluated by means of a caloric test and a Romberg rate. For the caloric test we performed bithermal caloric irrigation of the external ears while monitoring with an electronystagmography. The maximum velocity of the slow component of nystagmus from each ear was determined and the quantitative data were analyzed with a standard formula for canal paresis (CP) described by Jonkees^9^. Normal value of CP should be 25% or less. The Romberg ratio was calculated using Gravicorder G-5000 (Anima Corp., Tokyo), a value exceeding 1.0 would indicate a greater amount of postural sway during eyes closed^10^.

### Statistical analysis

Descriptive statistics are presented as the mean ± standard deviation and were compared using Welch’s two-sample t-test. The proportion of patients with each parameter was compared using Fisher’s exact test. Probability values of p < 0.05 were considered to be statistically significant. To evaluate whether clinical characteristics were associated with FHI, we performed a multivariable logistic regression analysis with significant factors (p < 0.05) from the univariate analysis. Statistical analysis was performed with free open-source software (R3.1.1; R Foundation for Statistical Computing; http://www.r-project.org). The datasets analyzed during the current study are available from the corresponding author on reasonable request.

## Results

### Findings of focal hyperintensity in the dorsal brain stem

**Figure 1:**
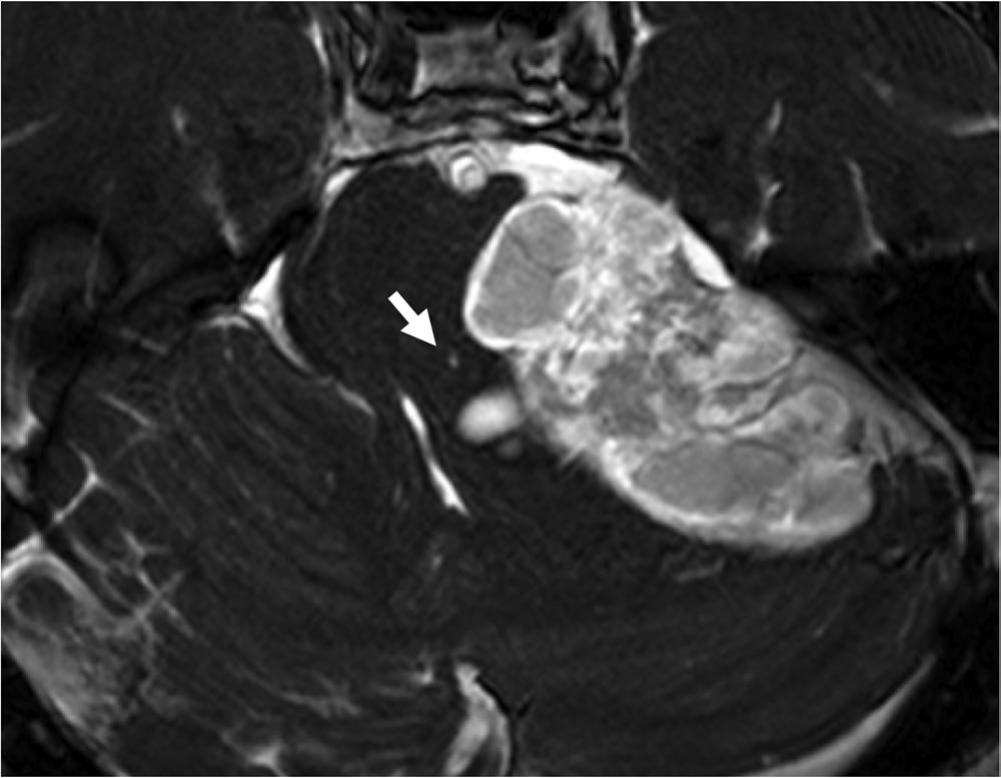
Representive MRI of the focal hyperintensity lesion (FHI) in the brain stem. Preoperative Gd-enhanced b-FFE MR image of a 64-year-old man shows a large cystic vestibular schwannoma at the left cerebellopontine cistern. A FHI can be observed clearly in the dorsal brain stem (arrow).

FHI in the dorsal brain stem was found in 25 (55.6%) of the patients with CPA tumors. Same results was obtained in both FFE and drive sequence. It was observed at a lateral angle of the fourth ventricle from the level of the inferior cerebellar peduncle to the mid-pons (Fig. 1). On 0.7mm-thick images 3–7 slices showed FHI. In many cases, FHI was observed as a tiny dot, but there were a few cases with FHI extending along a line from the posteromedial (upper limit) to the anterolateral (lower limit) dorsal brain stem. Among 25 patients with FHI, 21/25 (84.0%) lesions involved a single LVNc, and remaining four lesions involved MVNc and LVNc (3/25, 12.0%). In one case, FHI involved IVNc (1/25, 4.0%).

**Figure 2:**
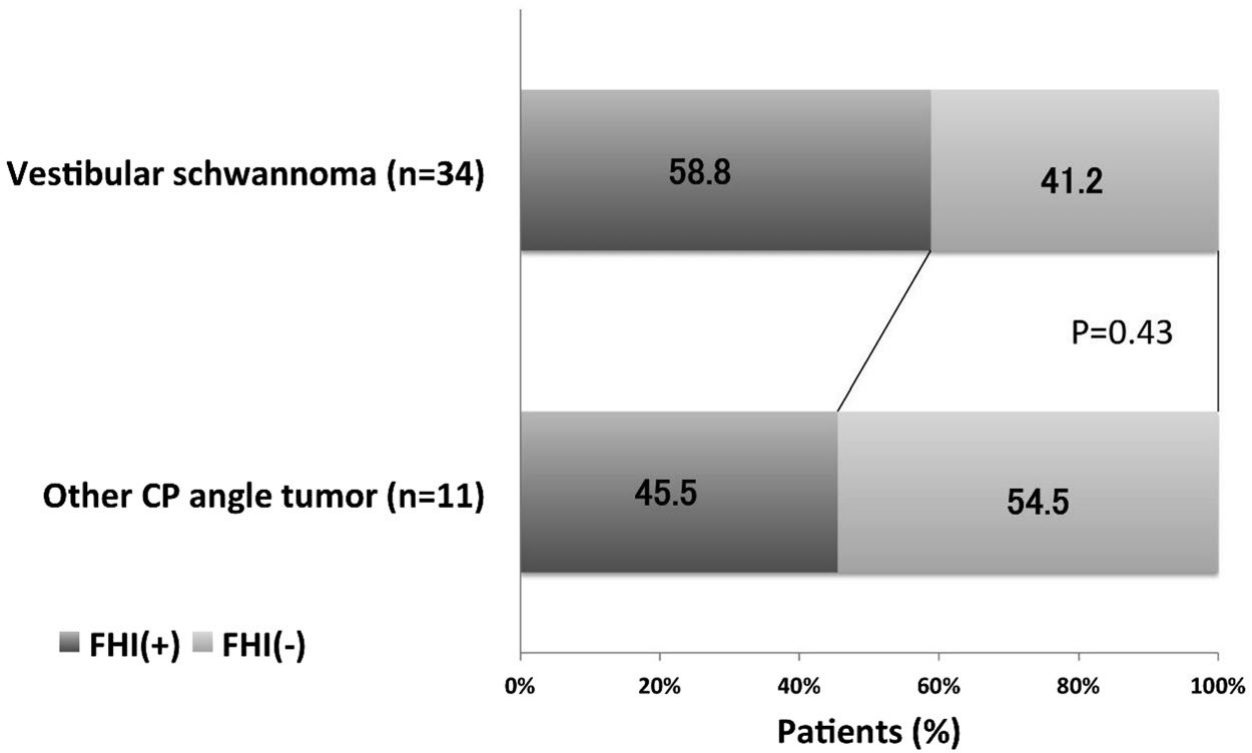
Comparison of frequency of FHI positivity between cases with vestibular schwannomas and other cerebellopontine angle tumors. There is no significant difference between two groups.

**Figure 3:**
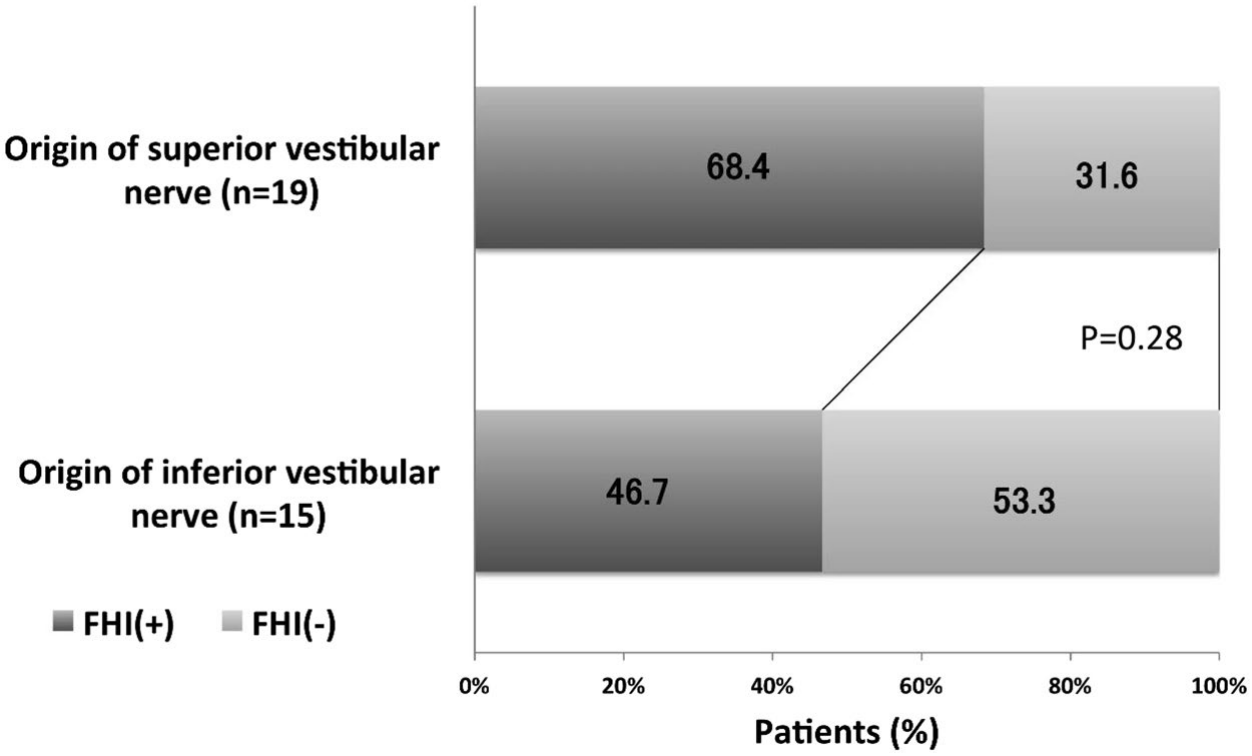
Comparison of frequency of FHI-positive cases based on nerve origin of tumors. There is no significant difference between two groups.

Among the 34 cases with vestibular schwannoma, 20 (58.8%) showed FHI, while FHI was observed in 5 (45.5%) (3 meningiomas, 2 facial nerve schwannomas) of the 11 other CPA tumors. There was no significant difference in frequency of FHI between the two groups of vestibular schwannomas and other CPA tumors (P = 0.43) (Fig. 2). Vestibular schwannoma originating from the superior vestibular nerve (SVN) was found in 19 cases and those originating from the inferior vestibular nerve (IVN) in 15 cases. FHI was observed in 13 (68.4%) of 19 cases of SVN origin and 7 (46.7%) in 15 cases of IVN (Fig. 3). The origin of the vestibular schwannomas did not correlate with the frequency of FHI (p = 0.28). In cases of five normal volunteers, FHI was not detected in the both side of dorsal brain stem in both 3D-drive and FFE sequences. In each control subject, all slices (35–50 slices) obtained from posterior fossa were also evaluated by two board-certified neurosurgeons and final decision was reached by consensus.

### Clinical characteristics and focal hyperintensity

Tables 1–3 shows the results of univariate and multivariate analyses of factors affecting the positive finding of FHI. Table 1 shows clinical characteristics of all the CPA tumors. In the univariate analysis, age (P = 0.011), max CPA (P = 0.019), hearing ability (p = 0.003) and CP (p = 0.002) of the patients had a significant effect on the presence of FHI. The multivariate regression analysis with significant these factors showed that only max CPA (odds ratio [OR], 3,23; 95% confidence interval [CI], 1.21–8.61; p = 0.019) was a significant factor affecting the FHI (the odds of max CPA are represented as odds per ten increase in size). Table 2 shows clinical characteristics of 34 vestibular schwannomas. In the univariate analysis, age (p = 0.025), max CPA (p < 0.001), hearing ability (p = 0.005) and CP (p < 0.001) of the patients showed a significant association with the occurrence of FHI. The multivariate regression analysis with significant these factors showed that only max CPA (OR, 9.03; 95% CI, 1.25–65.37; p = 0.029) was a significant factor affecting the FHI. Table 3 shows clinical characteristics of other CPA tumor. Both univariate and multivariate regression analyses showed that there were no significant factors affecting the presence of FHI (multivariate analysis was conducted with same factors in Tables 1 and 2).

**Table 1:** Summary of all patients with CPA tumor.

	Univariate			Multivariate	
	FHI (+) (n = 25)	FHI (−) (n = 20)	*p* value	Odds ratio (95% CI)	*p* value
Age (years)	53.6 ± 12.9	43.3 ± 13.1	0.011*	1.28 (0.67–2.45)	0.461
Max CPA (mm)	32.6 ± 10.0	22.3 ± 16.4	0.019*	3.23 (1.21–8.61)	0.019*
Hearing (dB)	52.8 ± 36.4	25.1 ± 20.5	0.003*	1.31 (0.94–1.83)	0.115
Canal paresis (%)	81.5 ± 26.0	48.9 ± 35.2	0.002*	1.07 (0.81–1.42)	0.636
Romberg rate	1.81 ± 0.65	1.61 ± 0.44	0.268	—	—

*CPA* cerebellopontine angle*, FHI* focal hyperintensity, *CI* confidence interval, *Max CPA* maximum diameter of the portion of the tumor within the cerebellopontine angle cistern, *dB* decibel, *p values lower than 0.05 were considered to be statistically significant, The odds of Max CPA are represented as odds per ten increase.

**Table 2:** Summary of patients with vestibular schwannoma.

	Univariate			Multivariate	
	FHI (+) (n = 20)	FHI (−) (n = 14)	*p* value	Odds ratio (95% CI)	*p* value
Age (years)	54.3 ± 13.4	44.9 ± 9.8	0.025*	1.95 (0.61–5.93)	0.239
Max CPA (mm)	32.0 ± 10.6	16.9 ± 9.9	<0.001*	9.03 (1.25–65.37)	0.029*
Hearing (dB)	59.1 ± 36.2	29.3 ± 21.6	0.005*	1.29 (0.85–1.96)	0.227
Canal paresis (%)	88.7 ± 19.3	45.8 ± 34.4	<0.001*	1.20 (0.78–1.87)	0.408
Romberg rate	1.89 ± 0.68	1.60 ± 0.47	0.184	—	—

*FHI* focal hyperintensity, *CI* confidence interval, *Max CPA* maximum diameter of the portion of the tumor within the cerebellopontine angle cistern, *dB* decibel, *p values lower than 0.05 were considered to be statistically significant, The odds of Max CPA are represented as odds per ten increase.

**Table 3:** Summary of patients with other CPA tumor.

	Univariate			Multivariate	
	FHI (+) (n = 5)	FHI (−) (n = 6)	*p* value	Odds ratio (95% CI)	*p* value
Age (years)	51.2 ± 11.7	39.7 ± 19.5	0.258	1.03 (0.26–4.04)	0.966
Max CPA (mm)	35.2 ± 7.2	34.8 ± 22.3	0.971	2.13 (0.15–29.72)	0.574
Hearing (dB)	27.8 ± 27.6	13.3 ± 11.6	0.325	3.26 (0.34–31.35)	0.306
Canal paresis (%)	52.8 ± 31.5	57.0 ± 40.1	0.859	0.51 (0.17–1.55)	0.235
Romberg rate	1.46 ± 0.40	1.63 ± 0.34	0.5561	—	—

*CPA* cerebellopontine angle*, FHI* focal hyperintensity, *CI* confidence interval, *Max CPA* maximum diameter of the portion of the tumor within the cerebellopontine angle cistern, *dB* decibel, *p values lower than 0.05 were considered to be statistically significant, The odds of Max CPA are represented as odds per ten increase.

### Postoperative changes of FHI

**Figure 4:**
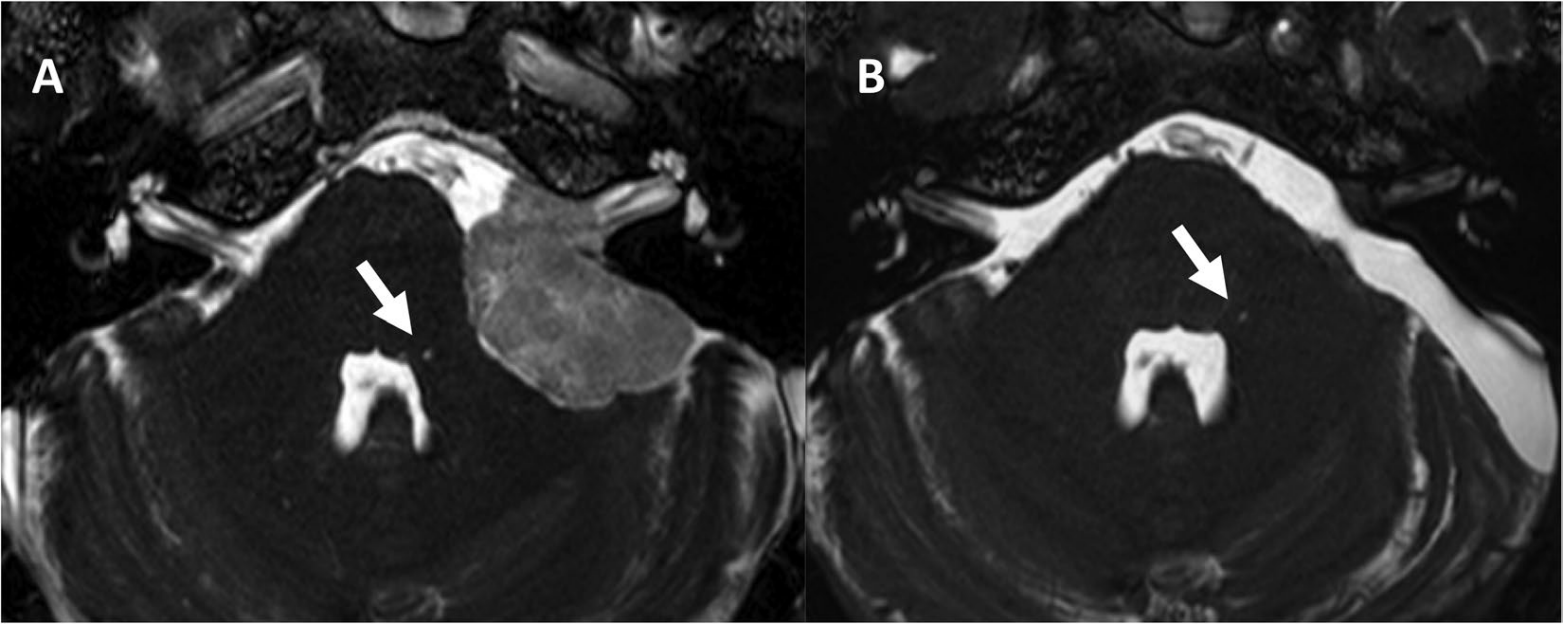
Representive preoperative Gd-enhanced b-FFE MR image of the FHI (arrow) in a patient with cerebellopontine angle meningioma (**A**). Postoperative (**B**) b-FFE image shows complete resection of tumor and no signal change of FHI (arrow).

We could examine postoperative 3 T images of only 7 cases because the routine postoperative follow-up study of the other patients used a 1.5 T scanner. Preoperative FHI was positive in 6 of the 7 cases. The mean interval between operation and postoperative MRI was 9.9 months. The follow-up MRI did not show any change in the FHI of the 6 cases. (Fig. 4A,B).

## Discussion

Okamoto *et al*. reported that some patients (14.6%) with vestibular schwannoma showed a tiny area of hyperintensity in the dorsal brain stem on T2-weighted images and that the location of this area coincided with the vestibular nuclear complex^4^. They concluded that, since this phenomenon was observed only in vestibular schwannomas more than 2 cm in size and not in patients with other kinds of CPA tumors, it indicates degenerations of the vestibular nucleus.

In our study, the frequency of FHI-positive cases (25/45 or 55.6%) was higher than reported by Okamoto *et al*. The reason for this discrepancy is likely to be the higher resolution of the MRI used in our study. The 3T-MR scanner provides a higher signal-to-noise ratio compared with the 1.5T-MR scanner used in the previous study, resulting in enhanced resolution and image quality^11^. About optimal sequence for the observation of FHI, we think 3D sequence was appropriate for the evaluation of tiny lesion less than 5 mm, because thin slice images obtained by conventional 2D-spin echo (SE) sequence decrease signal-to-noise ratio (SNR). Two different 3D sequence (i. e., drive and FFE) showed the same results in the patients. In this study, FHI was not detected in the contralateral dorsal brain stem in patients with FHI and not in both 3D-drive and FFE sequences of five normal volunteers (data not shown). Thus FHI is neither artifact nor sequence originating unexpected signal^4^. FHI would appear in the same side of dorsal brain stem in CPA tumors. Our results showed FHI is not a sign of specific pathology. We detected FHI of the dorsal brain stem not only in vestibular schwannomas, but also in other CPA tumors (3 meningiomas and 2 facial nerve schwannomas). The FHI was observed in cases of various CPA tumors with the similar frequency, the FHI could not be specific to vestibular schwannomas.

Vestibular schwannoma is by far the most frequent tumor among CPAs, accounting for 70% to 80%, and meningioma is the second most frequent, representing 10% to 15% of all CPA masses^1,2^. Facial schwannoma, on the other hand, is rare and difficult to diagnose before surgery^12^, which may be the reason why no occurrence of FHI in facial nerve schwannomas was reported in the previous study.

The vestibular nuclear complex consists of four separate nuclei, each receiving afferent information from several different sources. The medial and superior vestibular nuclei receive information directly from the cristae of the semi-circular canals. In addition, the vestibular nuclei receive sensory input from the otolith organs, from proprioceptors, particularly from the neck, as well as visual information^5^. The vestibular nuclear complex is located at the most lateral aspect of the floor of the fourth ventricle, but the normal vestibular nuclear complex is difficult to visualize directly on MR images^13–15^. Several prior studies have identified Wallerian degeneration as hyperintense areas on T2-weighted MR images^16,17^, while FHI is said to be most likely due to degeneration of vestibular nuclear complex^4^ as its location coincides anatomically with the vestibular complex. Our results using anatomical approach showed 84% of FHI involved LVNc, some of the cases in our study showed FHI extending along a line from the posteromedial to the anterolateral dorsal brain stem. We believe that the linear shape of the FHI might be due to Wallerian degeneration of the vestibular nerve itself rather than the vestibular nucleus. On the other hand, our results of clinical features affecting the positive FHI did not show significant factors except for the maximum size in the CP angle cistern in patients with vestibular schwannoma. Our hypothesis was that the degeneration of nucleus would be detected by neurophysiological approach, however, the positive findings of FHIs were not related to both the vestibular and cochlear function in all patients with CPA tumor. Although we did not conclusive explanation for the reason why the FHI is appeared in the patients with CPA tumor, our anatomical approach support that the presence of FHI probably indicates the degeneration of vestibular nucleus and nerves from which tumor originate. Because the size of tumor in the CPA cistern was an only factor influencing the positive FHI, the nerve compression also played an important role to form the FHI. From the point of clinical application of our results to daily clinical practice, the accurate meanings of FHI in MRI still have not been unclear. Our novel finding was that the ratio of FHI positive findings was higher than the previous report and this could not be a specific finding of vestibular schwannoma, thus clinicians should not make a misdiagnosis. We should continue to evaluate this finding as a predictor of tumor growth and surgical outcome such as cranial nerve preservation.

There are several limitations in our study. First, we evaluated which nucleus was involved among four vestibular nuclei based on the comparison between MR images and anatomical atlas slice-by-slice, however, distortion of the brain stem by tumor compression made it difficult to obtain reproducibility result. In cases with large tumor, we used postoperative imaging for comparison and final decision was made by inter-observer consensus. Further investigation and another approach were needed for the validation of our study, such as computational auto image fusion or superimpose on the atlas. Second, there were few data of postoperative follow-up images using 3T-MRI because most postoperative examinations are performed with a 1.5 T scanner. If the FHI developed only postoperatively, various causes would be proposed for the lesion, such as an infarction resulting from intraoperative vascular injury, direct surgical injury to the brainstem, or secondary degeneration of the injured cranial nerves.

Furthermore, examinations should be extensively done in patients with other pathology than tumors, for example with vestibular neuronitis. It would be then interesting if FHI positivity is found in them, as it also causes degeneration of the vestibular nerve.

In conclusion, focal hyperintensity (FHI) in the dorsal brain stem was observed more frequently than previously reported on the T2-weighted 3T-MR images of patients with CPA tumors. We detected FHI in cases with CPA tumors other than vestibular schwannomas, so that FHI is not specific to vestibular schwannomas as previously believed. In vestibular schwannomas, the presence of FHI was clinically affected by multiple factors and FHI is thought to indicate degeneration of the vestibular nerve and nuclei.
